# Sporoderm-Broken Spores of *Ganoderma lucidum* Sensitizes Ovarian Cancer to Cisplatin by ROS/ERK Signaling and Attenuates Chemotherapy-Related Toxicity

**DOI:** 10.3389/fphar.2022.826716

**Published:** 2022-02-21

**Authors:** Kaili Cen, Ming Chen, Mengye He, Zhenhao Li, Yinjing Song, Pu Liu, Qi Jiang, Suzhen Xu, Yunlu Jia, Peng Shen

**Affiliations:** ^1^ Department of Medical Oncology, The First Affiliated Hospital, Zhejiang University School of Medicine, Hangzhou, China; ^2^ Zhejiang Shouxiangu Botanical Drug Institute Co., Ltd., Hangzhou, China; ^3^ Department of Dermatology and Venereology, Sir Run Run Shaw Hospital, Zhejiang University School of Medicine, Hangzhou, China; ^4^ Department of Pathology, The First Affiliated Hospital, Zhejiang University School of Medicine, Hangzhou, China

**Keywords:** sporoderm-broken spores of *Ganoderma lucidum*, ganoderic acid D, ovarian tumor, chemosensitivity, adverse effect, reactive oxygen species, ERK signaling

## Abstract

Although platinum-based chemotherapeutics such as cisplatin are the cornerstone of treatment for ovarian cancer, their clinical application is profoundly limited due to chemoresistance and severe adverse effects. Sporoderm-broken spores of *Ganoderma lucidum* (SBSGL) have been reported to possess antitumor effects. However, the function and mechanism of SBSGL and its essential composition, ganoderic acid D (GAD), in the cisplatin therapy on ovarian cancer have yet to be investigated. Here, we investigated the combined effect of SBSGL and cisplatin in an ovarian tumor xenograft model. The results showed that combining SBSGL with cisplatin reduced tumor growth and ameliorated cisplatin-induced intestinal injury and myelosuppression. We also confirmed that GAD could enhance the therapeutic effect of cisplatin in SKOV3 and cisplatin-resistant SKOV3/DDP cells by increasing the intracellular reactive oxygen species (ROS). Mechanistically, we proved that ROS-mediated ERK signaling inhibition played an important role in the chemo-sensitization effect of GAD on cisplatin in ovarian cancer. Taken together, combining SBSGL with cisplatin provides a novel therapeutic strategy against ovarian cancer.

## Introduction

Ovarian cancer is one of the most malignant gynecological cancers with a 5-year survival rate of only about 48% (Kuroki and Guntupalli, 2020). For advanced-stage ovarian cancer, debulking surgery combined with platinum-based chemotherapy is still the main treatment strategy (Matulonis et al., 2016). Although 70% of patients initially respond to platinum-based chemotherapy, most of them suffer severe adverse effects and inevitably relapse because of chemoresistance (Ushijima, 2010). Therefore, enhancing the sensitivity of ovarian cancer to chemotherapy and reducing related side effects are expected to prolong the survival and improve the quality of life for patients with ovarian tumors.

Platinum-based chemotherapy is the standard first-line treatment for advanced ovarian tumors. Cisplatin is widely used in clinic practice as one of the most important platinum drugs. In this mechanism, cisplatin binds to nuclear DNA and generates a DNA lesion followed by a DNA damage response and mitochondria-mediated apoptosis (Galluzzi et al., 2012). On the other hand, cisplatin induces oxidative stress by interacting with cytoplasmic endogenous nucleophiles such as glutathione and methionine (Galluzzi et al., 2012). In addition, cisplatin accumulates in mitochondria and forms adducts with mitochondrial DNA, resulting in mitochondrial dysfunction and the production of reactive oxygen species (ROS) (Marullo et al., 2013). Oxidative stress further aggregates the injury of DNA and other organelles, such as mitochondria and endoplasmic reticulum, leading to cell cycle arrest and cell death eventually (Cui et al., 2018). However, tumors resistant to cisplatin can circumvent cisplatin-induced death through multiple mechanisms, one of which is the regulation of redox homeostasis. Cisplatin-resistant ovarian tumors contained less mitochondrial content and lower mitochondrial ROS (Kleih et al., 2019). Moreover, the cisplatin-resistant tumor showed upregulated expression of various antioxidative genes, such as the superoxide dismutase (SOD) and nuclear factor E2-related factor 2 (NRF2), to balance the intracellular redox state (Kim et al., 2019). Therefore, readjusting the redox balance and tilting tumor cells to oxidative stress may provide a new strategy to overcome cisplatin resistance.

The medicinal mushroom *Ganoderma lucidum* has been widely used in Asian countries for more than 2000 years and possesses many functions such as anti-inflammation, immune regulation, and antidiabetic and antitumor effects (Bishop et al., 2015). In the past, the fruit body and mycelia of *Ganoderma lucidum* were considered the major executors of the above bioactivities (Kuo et al., 2006; Oliveira et al., 2014). However, with the development of wall-breaking and phytochemical techniques, various constituents of *Ganoderma lucidum* spores have been extracted and identified. Among them, triterpenoids and polysaccharides are currently the most widely studied and considered as the main active ingredients of sporoderm-broken spores of *Ganoderma lucidum* (SBSGL) (Lin and Yang, 2019). There is strong evidence demonstrating that SBSGL has a broad spectrum of bioactivities such as the antitumor effect in osteosarcoma, cholangiocarcinoma, and colorectal tumors (Li et al., 2016; Li et al., 2017; Zhang et al., 2019); immunomodulation (He et al., 2020); and protective effect on radiotherapy- or chemotherapy-induced toxicity (Dai et al., 2019; Li et al., 2020). However, whether SBSGL could enhance the antitumor effect of cisplatin in ovarian cancer and ameliorate cisplatin-induced side effects still waits to be verified. In addition, many monomers have been isolated from SBSGL, and their therapeutic value has been widely studied in different disease models. Among them, ganoderic acid D (GAD), a highly oxygenated tetracyclic triterpenoid (Cheng et al., 2013), is a major component of triterpenoids from SBSGL (Liu et al., 2011). Previously, GAD was reported to have an antitumor effect in esophageal squamous carcinoma by inducing ROS-dependent apoptosis (Shao et al., 2020). However, few studies have reported the effect of GAD on ovarian cancer and its effects on redox regulation.

In this study, we examined the potential sensitizing effect of SBSGL on the therapeutic effect of cisplatin on ovarian cancer in a xenograft model. We demonstrated that combining SBSGL with cisplatin could enhance the chemosensitivity of ovarian cancer cells and attenuate cisplatin-induced intestinal injury and myelosuppression in nude mice transplanted with ovarian cancer cells. Furthermore, we proved the synergistic antitumor effect of GAD with cisplatin in cisplatin-sensitive and cisplatin-resistant ovarian cancer cell lines. Mechanistically, we found that combining cisplatin with GAD could upregulate oxidative stress and subsequently inhibit the activation of the ERK signaling pathway, eventually leading to the suppression of cell proliferation, induction of apoptosis, and reversal of cisplatin resistance.

## Materials and Methods

### Chemical Reagents

Sporoderm-broken spores of *Ganoderma lucidum* (SBSGL) were provided by Zhejiang Shouxiangu Pharmaceutical Co., Ltd. Ganoderic acid D (HY-N1511, purity >99%), cisplatin (HY-17394), and the ERK agonist, LM22B-10 (HY-104047) were purchased from MedChemExpress (Shanghai, China). Crystal violet (C0121) and N-acetyl-L-cysteine (NAC, S0077) were purchased from Beyotime (Shanghai, China). Primary antibodies we used were JNK (9252), p-JNK (4668), ERK1/2 (4695), p-ERK (4370), p38 (8690), and p-p38 (4511), and all the above antibodies were purchased from Cell Signaling Technology (Danvers, MA, United States).

### Sample Preparation and Storage

SBSGL is a commercially available food supplement approved by the State Administration for Market Regulation (SAMG) in China. In brief, intact *Ganoderma lucidum* spores (raw materials) were first subjected to supersonic air jet milling to break the sporoderm, and then extracted by water twice, at ten-fold and eight-fold volumes, respectively. The combined solution was filtered, concentrated, and dried to obtain the SBSGL used in this work. The yield of SBSGL was approximately 10% by mass. As for the preparation of the test solution used in the animal experiment, SBSGL was reconstituted in ddH_2_O at 0.2 g/ml and the mixture was heated in boiling water for 20 min. Since the mixture was a suspension, the SBSGL was immediately mixed upside down and aliquoted. Finally, the stock was stored at −20°C and used up in 1 week.

### Animals and Treatment

Female BALB/c nude mice (18 ± 2 g, 6 weeks old) were purchased from Hangzhou Medical College (Hangzhou, China, certificate no. 20210705Abzz0100018725). The study was approved by the Institutional Animal Care and Use Committee, Zhejiang Center of Laboratory Animals (license number: ZJCLA-IACUC-20050017). All mice were kept in a specific pathogen-free environmental condition, allowing free access to water and food. After 1 week for adaption, each mouse was injected with 0.1 ml of cell suspension containing 4 × 10^6^ SKOV3 into the right flanks. When tumor volumes reached approximately 100 mm^3^, 20 mice were divided randomly into four groups (*n* = 5 in each group). They received corresponding treatment as follows: 1) Control group: mice were orally administered (i.g.,) daily and intraperitoneally injected (i.p.,) every week with an equal volume of saline with other groups. 2) SBSGL group: mice were orally administrated with SBSGL (2 g/kg) every day. 3) Cisplatin group: mice were intraperitoneally injected with cisplatin (3 mg/kg) every week. 4) SBSGL + Cisplatin group: SBSGL and cisplatin were administrated according to the aforementioned regimes, and mice were pretreated with SBSGL for 3 days. Previous literature was referred to determine the action concentration range of SBSGL, and the final dose was calculated by its clinical recommended dose (4–8 g/kg per day for an adult) based on the body surface area (BSA) normalized method (Liu et al., 2002; Reagan-Shaw et al., 2008; Chen et al., 2016). Bodyweight and tumor volume were measured every 3 days. The tumor volume was calculated as follows:

$$\text{Tumor}\;\text{volumes}\;\left(\text{V}\right)=\frac{1}{2}\times \text{length}\times {\text{width}}^{2}.$$ (1)

Finally, all mice were sacrificed with 2% isoflurane. The blood samples were collected for the kidney function test and hematological evaluation. For further histopathological analysis, organs that are susceptible to cisplatin, such as kidneys, duodenum, thighbones, and tumors, were collected.

### Cell Lines and Culture Conditions

Human ovarian tumor cell line SKOV3 was obtained from the American Type Culture Collection (ATCC). Cisplatin-resistant cell line SKOV3/DDP was generously provided by Professor Weiguo Lv, Women’s Hospital School of Medicine, Zhejiang University. Both SKOV3 and SKOV3/DDP were cultured in McCoy’s 5A medium supplemented with 10% fetal bovine serum. All cells were maintained at 37°C in a humidified incubator with 5% CO_2_.

### Kidney Function Tests

Serum samples were collected by centrifugation at 4000 rpm for 10 min. Then the levels of blood urea nitrogen (BUN) and serum creatine (SCR) were measured by a chemical analyzer (LW C400, Landwind, Shenzhen, China).

### Hematological Evaluation

Blood was obtained from mice *via* the abdominal aorta and preserved in a micropipette coated with K_3_EDTA. Cells were analyzed by an automatic hematology analyzer (BC-5000VET, Mindray, Shenzhen, China). The parameters of blood cells included red blood cells (RBC), hemoglobin (Hb), white blood cells (WBC), neutrophils, platelets (PLT), monocytes, eosinophils, and basophils.

### Histology Analysis

Kidneys, duodenum (Hu et al., 2021), and thighbones were collected for histological analysis. All tissues were immersed in 4% paraformaldehyde at room temperature for more than 24 h. Thighbones were decalcified by EDTA decalcification fluid. Then, all the tissues were dehydrated and embedded in paraffin. About 4 μm thick of paraffin-embedded tissues were sectioned and stained with hematoxylin–eosin (H&E). Finally, the sections were observed by an upright optical microscope (Nikon Eclipse E100, Japan). To evaluate the kidney injury, tubular damage was scored based on the percentage of the damaged area of the tubule epithelial cells: 0-normal, 1- <10%, 2-10–25%, 3-26–75%, and 4->75%. Tubular epithelial damage was defined as degeneration, atrophy, necrosis, and intraluminal aggregation of cells and proteins, as well as hyperemia and inflammatory cell infiltration of the mesenchyme (Meng et al., 2017). In addition, intestinal injury was determined based on morphological changes of villi, crypts, gland destruction, and lamina propria atrophy (Wu et al., 2019; Zhang et al., 2020). The histological evaluation was performed in a blind manner.

### Ki-67 Staining and TUNEL Assay

The paraffin-embedded tumor specimens were sectioned into 4 μm slides. To evaluate the expression of Ki-67, slides were blocked and incubated with the antibody targeting Ki-67 (Servicebio, GB111499, 1:300) at 4°C overnight. The next day, slides were incubated with the corresponding second antibody at room temperature for 50 min. Finally, slides were visualized with DAB substrate buffer (DAKO, K5007) and photographed using a light microscope at a magnification of x 400. In addition, the TUNEL apoptosis detection kit (YEASEN, 40306ES20) was used to measure the extent of apoptosis in tumors according to the manufacturer’s instructions. Ortho-Fluorescent Microscopy (Nikon Eclipse C1, Japan) was used to observe the nuclear expression of TUNEL-positive cells at a magnification of x200. Both the ki-67 positive rate and the apoptosis rate were calculated by counting the number of positive cells/total cells in five fields randomly selected with ImageJ software.

### CCK-8 Assay

Cell viability was measured using Cell Counting Kit-8 (CCK-8, Meilunbio, Dalian, China). Cells were seeded in 96-well plates at a density of 8000 per well. After 24 h for adherence, cells were treated with different concentrations of cisplatin and GAD alone or in combination for 24 h. To determine the role of ROS, cells were exposed to NAC (10 mM), GAD + cisplatin, and NAC + GAD + cisplatin for 24h, respectively. To explore the role of p-ERK in the combined treatment, both SKOV3 and SKOV3/DDP were pretreated with 20μM LM22B-10, an ERK agonist for 6h, followed by GAD + cisplatin for 24 h. At the end of the treatment, the medium was replaced by 100 μl fresh medium containing 10% CCK-8 and the cells were incubated at 37°C for 1.5 h. The optical density (OD value) was measured by the microplate reader (Varioskan Flash, Thermo Scientific, America) at a wavelength of 450 nm. Cell viability relative to control was calculated as follows:

$$\text{Cell}\;\text{viability}\;\left(\text{\%}\right)=\frac{\text{OD}\left(\text{treated}\right)-\text{OD}\left(\text{blank}\right)}{\text{OD}\left(\text{control}\right)-\text{OD}\left(\text{blank}\right)}\times 100\text{\%}\text{.}$$ (2)

The half-maximal inhibitory concentration (IC50 value) was calculated by SPSS 25.0 software.

### Colony Formation Assay

SKOV3 and SKOV3/DDP were exposed to indicated treatments for 24 h in 12-well plates and subsequently re-plated into new plates at a density of 600 per well. Ten days later, cells were fixed with 4% paraformaldehyde and stained with crystal violet. The images were photographed, and colonies with more than 50 cells were counted under the microscope.

### Flow Cytometry

To detect apoptosis of cells, the Annexin V-FITC/PI apoptosis kit (70-AP101-100, Multi Sciences, Hangzhou, China) was used according to the protocol. In brief, after indicated treatments, the supernatant and cells were collected by digestion with trypsin without EDTA. Then, cells were resuspended with 200 μl 1x binding buffer, containing 5 μl Annexin V-FITC and 10 μl PI. Samples were gently mixed and incubated at room temperature for 10 min in the dark. Finally, cells were detected by the flow cytometer (ACEA NovoCyteTM, ACEA Biosciences, America). To measure the level of intracellular ROS in cells, a DCFH-DA fluorescent probe (S0033S, Beyotime, Shanghai, China) was used according to the instructions. After treatments, cells were washed twice with PBS and incubated in the serum-free medium containing 5 μM DCFH-DA for 20 min at 37°C in the dark. Finally, cells were washed twice by PBS and collected for analysis by the flow cytometer.

### Western Blotting

The cells or tissues were lysed with NP-40 lysis buffer (P0013F, Beyotime, Hangzhou, China) supplemented with 1 mM PMSF (TS505, Beyotime) as well as a protease inhibitor cocktail (Sigma-Aldrich). The protein concentration of samples was measured by the BCA assay (23227, Thermofisher). An equal amount of protein from the samples was subjected to SDS-PAGE and transferred to PVDF membranes (Millipore). Immunoblots were blocked with 5% defatted milk for 1 h and then incubated with the corresponding primary antibodies at 4°C overnight. After washing with TBST three times, membranes were incubated with the corresponding second antibodies for 2 h at room temperature. Finally, protein bands were visualized by a Pierce Chemiluminescence ECL kit (34577, Thermofisher), and the grayscale value was quantified by ImageJ software.

### DHE Staining

To measure the level of ROS in tumor tissues, DHE staining was performed according to previous research (Wang et al., 2021). In brief, tumor tissue sections were dewaxed, hydrated, and immersed in the 3% H_2_O_2_ solution for 15 min, 80% alcohol for 30 min, and then washed with PBS three times. Next, sections were blocked and incubated with 20 μM DHE solution (Beyotime, China) for 10 min at room temperature, followed by washing with PBS. Finally, sections were stained with DAPI and photographed by a confocal laser microscope (Nikon, Tokyo, Japan).

### Statistical Analysis

All data were exhibited as the mean ± SD or SEM of the biological replicates. Statistical analysis was performed with Prism 8. One-way ANOVA followed by Tukey’s multiple comparison test or two-way ANOVA was used to compare data among different groups. ^***^ *p* < 0.001, ^**^ *p* < 0.01, and ^*^ *p* < 0.05 were considered statistically significant.

## Results

### Sporoderm-Broken Spores of *Ganoderma Lucidum* Sensitized Ovarian Cancer to Cisplatin *In Vivo*

**FIGURE 1:**
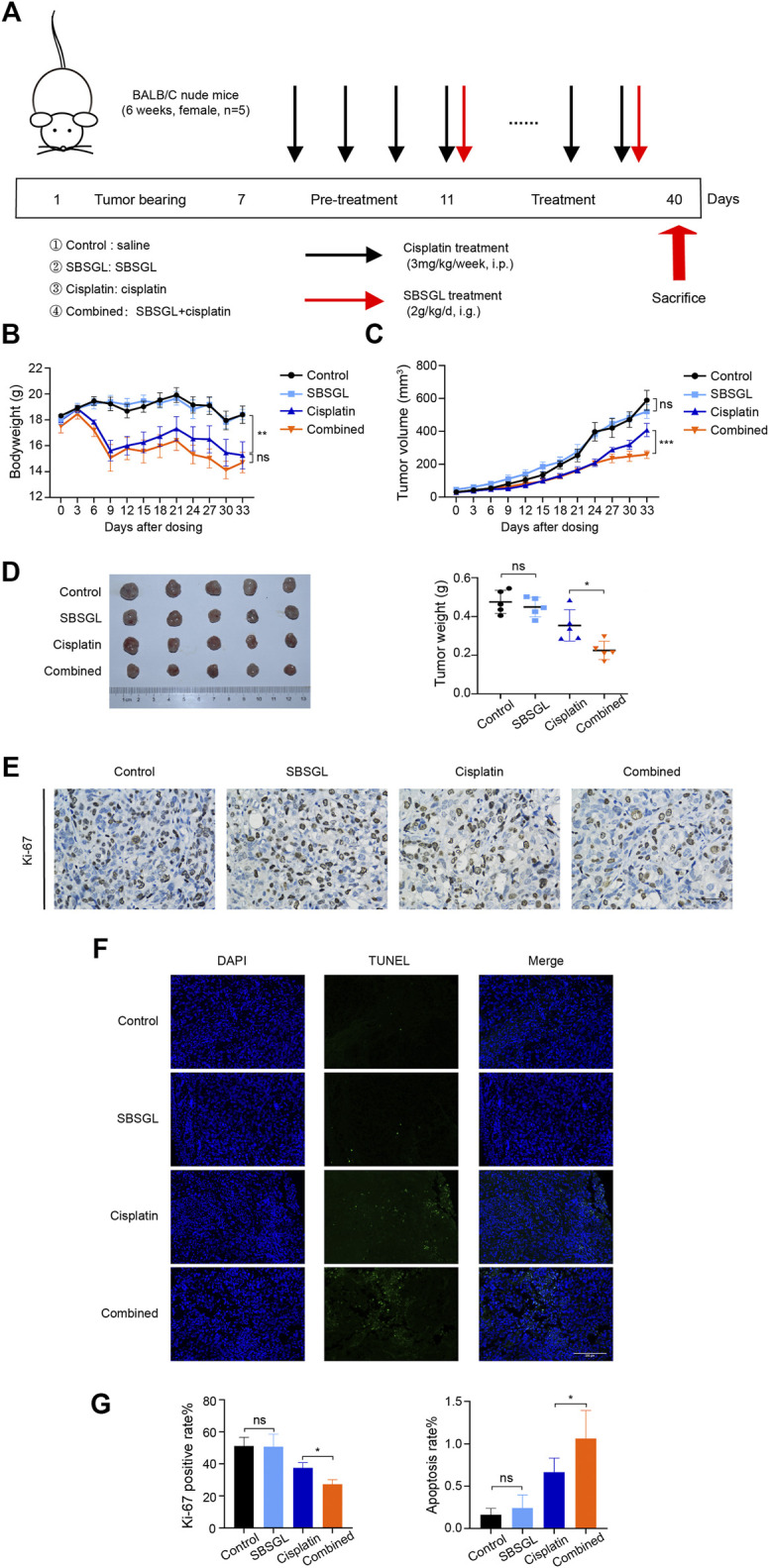
Sporoderm-broken spores of *Ganoderma lucidum* (SBSGL) sensitized ovarian tumor to cisplatin *in vivo*. **(A)** The scheme of the animal experiment. **(B)** The changes of bodyweight of nude mice. **(C)** The changes of tumor volume. **(D)** The picture of tumor masses and quantitative analysis of tumor weight. **(E)** The expression of Ki-67 in tumor tissues. **(F)** TUNEL-positive nucleus (green) indicated cell apoptosis. Cell nuclei were detected by DAPI (blue). **(G)** The quantitative analysis of positive rate of Ki-67 and TUNEL. Data are presented as the mean ± SD or SEM (*n* = 5). Compared to the control group or the cisplatin group: ^***^ *p* < 0.001, ^**^ *p* < 0.01, ^*^ *p* < 0.05, ns, no statistical significance.

To evaluate the sensitization effect of SBSGL on cisplatin, we first established a subcutaneous ovarian tumor xenograft model, and the nude mice were treated with SBSGL, cisplatin, or their combination (Figure 1A). The results showed that the bodyweight of mice in the cisplatin group was obviously reduced relative to the control but had no significant difference from those in the combined treatment (Figure 1B), implying that SBSGL would not aggravate cisplatin-induced weight loss. Meanwhile, compared with the single cisplatin treatment, the combined treatment of cisplatin and SBSGL further enhanced the inhibitory effects of cisplatin on tumor volume and tumor weight (Figures 1C, D). Next, we exfoliated tumors and performed Ki-67 and TUNEL staining. The results showed that tumors from the combined treatment group showed a significantly decreased level of Ki-67 and exhibited higher fluorescence intensity of TUNEL than those of the cisplatin group, suggesting that combining SBSGL with cisplatin further repressed the proliferation and promoted apoptosis of ovarian tumors *in vivo* (Figures 1E–G).

### Sporoderm-Broken Spores of *Ganoderma lucidum* Attenuated Cisplatin-Induced Intestinal Injury and Myelosuppression *In Vivo*

Common adverse effects of cisplatin include nephrotoxicity, gastrointestinal toxicity, and myelosuppression (Qi et al., 2019). First, to explore the kidney protection effect of SBSGL, we performed HE of the kidneys and evaluated tubular damage scores. The kidneys of mice treated with cisplatin or in combination with SBSGL showed obvious edema and atrophy of renal tubular epithelial cells along with inflammatory cell infiltration in the mesenchyme (Supplementary Figure S1A). Meanwhile, the tubular damage scores of the two groups had no significant difference (Supplementary Figure S1A). In addition, we tested serum creatinine (SCR) and blood urea nitrogen (BUN) in different groups, which are hallmarks of kidney function. The results showed that cisplatin treatment increased the level of SCR and BUN in the blood but had no difference from the combined treatment (Supplementary Figure S1B). The results above implied that SBSGL had no obvious protective effect on cisplatin-induced nephrotoxicity.

**FIGURE 2:**
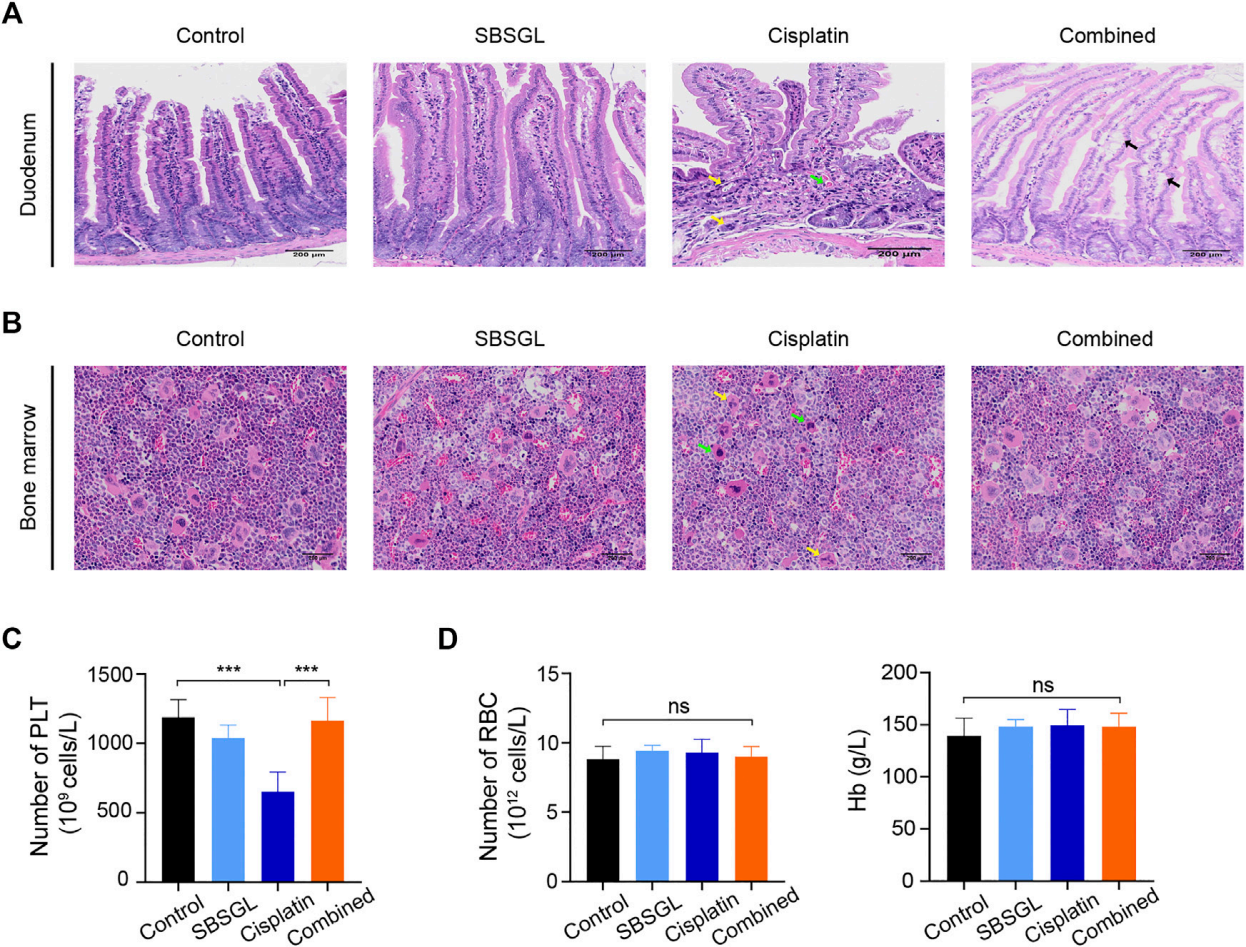
SBSGL ameliorated cisplatin-induced intestinal injury and myelosuppression. **(A)** The morphological changes of duodenum. Arrows indicate atrophy of glands (yellow), interstitial hyperemia (green), and edema (black) of villus. **(B)** The histological structures of bone marrow in thighbones of nude mice. Arrows indicate the increasing proportion of unsegmented megakaryocytes (green) and cytoplasmic atrophy (yellow). **(C,D)** The number of PLT and RBC, and the content of Hb in blood of nude mice. PLT: platelets; RBC: red blood cells; Hb: hemoglobin. Data are presented as the mean ± SD (*n* = 3). ^***^ *p* < 0.001; ns, no statistical significance.

Next, we evaluated the intestinal injury of mice with different treatments. The duodenum in the cisplatin group showed a disordered structure of villi, gland atrophy, as well as lamina propria hyperemia (Figure 2A); supplementing SBSGL with cisplatin could partially attenuate these intestinal damages as the duodenum of the combined group only exhibited slight edema of villi (Figure 2A).

Finally, to evaluate hematopoiesis in the bone marrow, we performed HE on thighbones from nude mice with different treatments. The results showed that although the bone marrow of four groups exhibited relatively normal hematopoiesis, the cisplatin treatment obviously reduced the proportion of segmented megakaryocytes and induced cytoplasmic atrophy of megakaryocytes, implying dysfunction of megakaryocyte differentiation (Figure 2B). However, combining SBSGL with cisplatin could recover the proportion of segmented megakaryocytes and normalize their morphology (Figure 2B). Furthermore, we counted various blood cells in peripheral blood. Consistent with previous HE results, cisplatin significantly reduced the number of platelets and SBSGL effectively attenuated cisplatin-induced platelet decrease (Figure 2C). However, the number of white blood cells (WBC), neutrophils, as well as monocytes had no difference among the four groups (Supplementary Figure S2), implying that granulocytes were not as susceptible as megakaryocytes to cisplatin. In addition, both cisplatin and SBSGL had no obvious cytotoxicity on erythroid cells (Figure 2D). Results from this part of our study thus indicate that combining SBSGL with cisplatin ameliorates cisplatin-induced megakaryocyte suppression.

### Ganoderic Acid D Enhanced the Effect of Cisplatin on the Ovarian Tumor *In Vitro*

**FIGURE 3:**
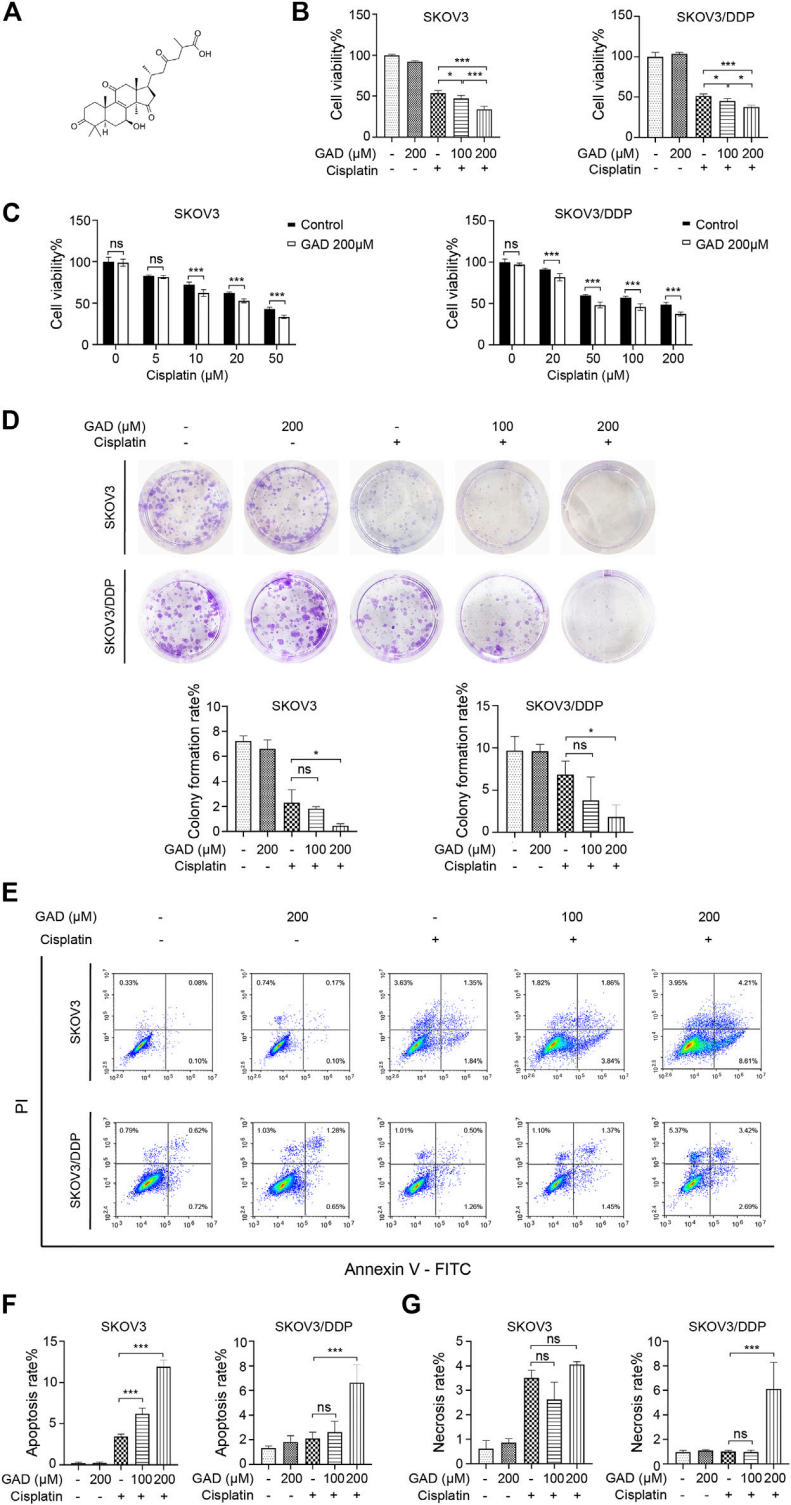
Ganoderic acid D (GAD) enhanced cisplatin-induced cytotoxicity in cisplatin-sensitive and cisplatin-resistant ovarian tumor cell lines. **(A)** The chemical structure of GAD. **(B)** The cell viability of SKOV3 and SKOV3/DDP treated with GAD and cisplatin (40 μM for SKOV3 and 200 μM for SKOV3/DDP) for 24 h. **(C)** The cell viability of SKOV3 and SKOV3/DDP exposed to 200 μM GAD and different concentrations of cisplatin for 24 h. **(D)** The images of colony formation and corresponding quantitative analysis. **(E–G)** The apoptosis and necrosis of SKOV3 and SKOV3/DDP and their corresponding quantitative analysis. Data are presented as the mean ± SD (*n* = 3). ^***^ *p* < 0.001; ns, no statistical significance.

To clarify which component of SBSGL had the sensitization effect on cisplatin in ovarian tumors, GAD, one of the main components of SBSGL, was selected for further research. The chemical structure of GAD is shown in Figure 3A. First, CCK-8 was used to test the effects of GAD and cisplatin on the viability of the cisplatin-sensitive SKOV3 cell line and the cisplatin-resistant SKOV3/DDP cell line. The results showed that GAD reduced the viability of SKOV3 and SKOV3/DPP in a time- and concentration-dependent manner (Supplementary Figure S3A). After 24 h of treatment, the IC50 values of cisplatin were 39.917 and 207.191 μM in SKOV3 and SKOV3/DDP, respectively (Supplementary Figure S3B). To explore whether GAD could enhance the cytotoxicity of cisplatin, the non-toxic concentration of GAD (200 μM for both SKOV3 and SKOV3/DDP) and IC50 values of cisplatin (40 μM for SKOV3 and 200 μM for SKOV3/DDP) at 24 h of 2 cell lines were chosen for the following experiments. The results showed that the combined treatment further decreased cell viability in both cell lines, and the inhibitory effect was positively related to the concentration of GAD (Figure 3B). Next, we exposed both cell lines to different concentrations of cisplatin with or without 200 μM GAD for 24 h. The results of CCK-8 showed that GAD could enhance cisplatin-induced proliferation inhibition in both SKOV3 and SKOV3/DDP cells (Figure 3C). Moreover, a colony formation assay, reflecting the degree of tumor malignancy, was performed to test the proliferation capability of tumors. The results showed that compared with the single cisplatin treatment, combining GAD with cisplatin could further inhibit the colony formation rate of SKOV3 (Figure 3D). A similar result was also observed in SKOV3/DDP cells (Figure 3D). Furthermore, to investigate cell death of SKOV3 and SKOV3/DDP, Annexin V/PI staining was used, and the results showed that the apoptosis rate of SKOV3 cells in the GAD + cisplatin group (12.82%) was 4 times higher than 3.19% in the cisplatin group. A similar trend was also observed in SKOV3/DDP cells (Figure 3E). The apoptosis rate of combined treatment was positively correlated with the concentration of GAD in SKOV3 but not in SKOV3/DDP cells (Figure 3F). Interestingly, it was worth noting that in SKOV3/DDP cells, the combined treatment of GAD and cisplatin evidently promoted other forms of cell death except apoptosis, as the combined treatment significantly increased the PI-single positive rate, which indicated necrosis of cells, compared with single cisplatin treatment (Figure 3G). Interestingly, the change in the PI single positive rate between GAD + cisplatin and the cisplatin group in SKOV3 was not as obvious as that in SKOV3/DDP cells (Figure 3G).

### Combining Ganoderic Acid D with Cisplatin Induced Reactive Oxygen Species-Mediated Cell Proliferation inhibition and Death

**FIGURE 4:**
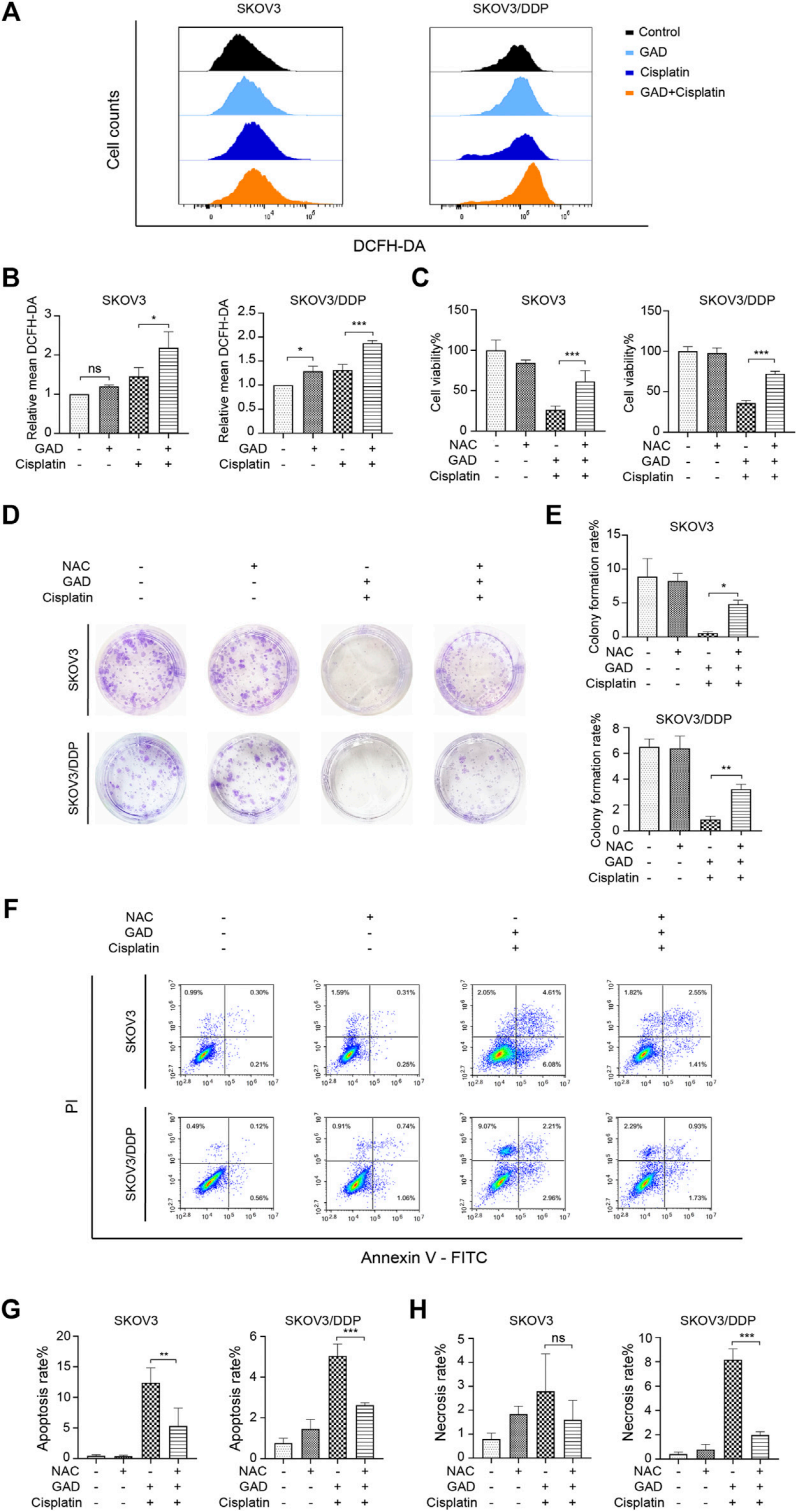
The combination of GAD and cisplatin induced ROS-mediated growth inhibition and cell death in SKOV3 and SKOV3/DDP cell lines. **(A)** SKOV3 and SKOV3/DDP were treated with GAD (200 μM) and cisplatin (40 μM for SKOV3 and 200 μM for SKOV3/DDP) alone and their combination for 24 h and the intracellular ROS was measured by DCFH-DA probe. **(B)** The quantitative analysis of mean inflorescence intensity of DCFH-DA in SKOV3 and SKOV3/DDP. **(C)** Supplementing with 10 mM NAC for 24 h partially reversed the cell viability inhibition in SKOV3 and SKOV3/DDP. **(D,E)** The images of colony formation and the corresponding quantitative analysis. **(F)** The apoptosis and necrosis of SKOV3 and SKOV3/DDP treated with the combination of GAD and cisplatin with or without 10 mM NAC for 24 h. **(G,H)** The quantitative analysis of apoptosis rate and necrosis rate of SKOV3 and SKOV3/DDP. NAC: N-acetyl-L-cysteine. Data are presented as the mean ± SD (*n* = 3). ^***^ *p* < 0.001, ^**^ *p* < 0.01; ns, no statistical significance.

To explore the mechanism of GAD-induced sensitization effect on cisplatin in ovarian tumors, DCFH-DA was used to test intracellular ROS levels by flow cytometry. The results showed that the combination of GAD and cisplatin induced higher ROS than single cisplatin treatment in both SKOV3 and SKOV3/DDP cells, while single GAD treatment only slightly increased ROS in SKOV3/DDP but not in SKOV3 (Figures 4A, B). As ROS plays a dual role in promoting cell survival or cell death (Aggarwal et al., 2019), here we explored the role of ROS in mediating the sensitization effect of GAD on cisplatin by using N-acetyl-l-cysteine (NAC), a ROS scavenger. The flow cytometry data showed that 10 mM NAC treatment for 24 h reduced ROS in the combined treatment of both SKOV3 and SKOV3/DDP cells (Supplementary Figure S4). Next, we performed CCK-8, colony formation assay, and Annexin V/PI staining on SKOV3 and SKOV3/DDP, which were exposed to the GAD + cisplatin with or without 10 mM NAC for 24 h. The results showed that cell viability and the colony formation rate were obviously reversed by NAC in both cell lines (Figures 4C–E). Consistently, the apoptosis rate was reduced in SKOV3 and SKOV3/DDP, and the cell necrosis was also reversed by NAC in SKOV3/DDP (Figures 4F–H). Taken together, results from this part of our study indicate that combining GAD with cisplatin inhibits tumor growth and promotes cell death *via* induction of ROS.

### Reactive Oxygen Species/ERK Signaling Pathway Mediated Sporoderm-Broken Spores of *Ganoderma lucidum* and Ganoderic Acid D-Induced Cisplatin Sensibilization in Ovarian Tumor

**FIGURE 5:**
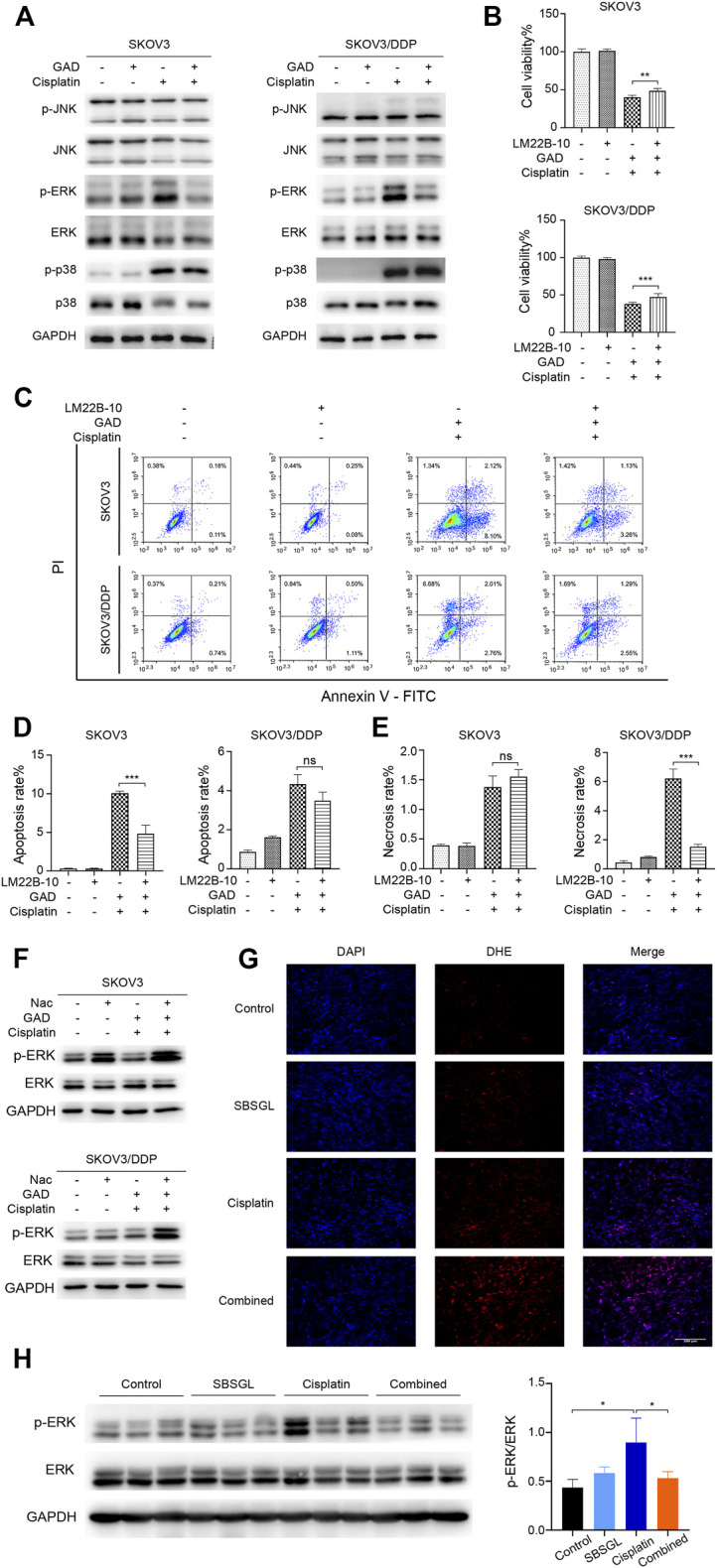
ROS/ERK signaling mediated the combination effect of GAD and cisplatin in SKOV3 and SKOV3/DDP. **(A)** The phosphorylated protein expression of MAPK signal pathways in SKOV3 and SKOV3/DDP treated with GAD (200 μM) and cisplatin (40 μM for SKOV3 and 200 μM for SKOV3/DDP) alone and their combination for 24 h. To explore the role of ERK signaling, SKOV3 and SKOV3/DDP were pretreated with the ERK activator, LM22B-10 (20 μM) for 6h, followed by the combination of GAD and cisplatin for 24 h. The cell viability **(B)** and death **(C)** were detected by the CCK-8 assay and Annexin V/PI assay. **(D,E)** The quantitative analysis of cell apoptosis and necrosis. **(F)** The protein expression of p-ERK in SKOV3 and SKOV3/DDP incubated with the combination of GAD and cisplatin with or without 10 mM NAC for 24 h. **(G)** The DHE fluorescence (red) in tumor tissues. Cell nuclei were detected by DAPI (blue). **(H)** The protein expression of p-ERK in tumor lysate. Data are presented as the mean ± SD (*n* = 3). ^***^ *p* < 0.001, ^**^ *p* < 0.01, ^*^ *p* < 0.05, ns, no statistical significance.

**FIGURE 6:**
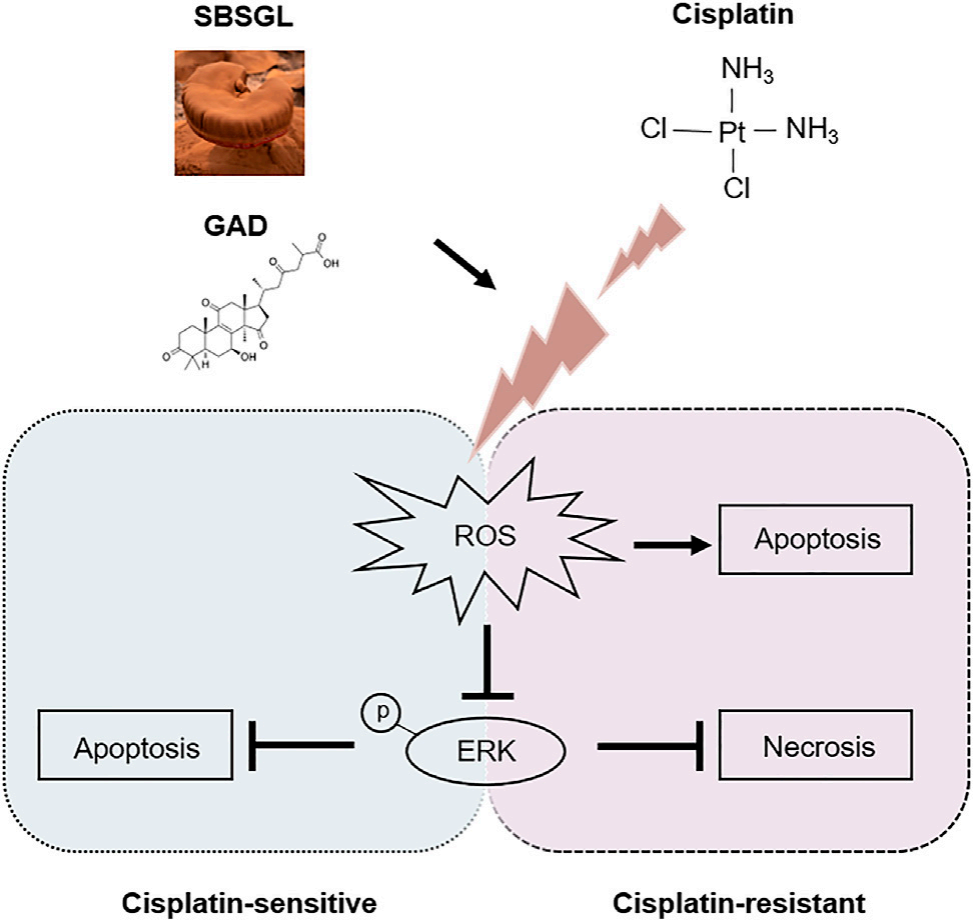
The underlying mechanism involved in the chemo-sensitization effect of SBSGL/GAD on ovarian cancer.

The mitogen-activated protein kinase (MAPK) signaling pathway is an important executor of the downstream effects of ROS and regulates various cellular processes like cell proliferation, differentiation, and apoptosis (Jalmi and Sinha, 2015). Therefore, we speculated that MAPK signaling might participate in the ROS-mediated sensitization effect of GAD on cisplatin. To verify our conjecture, Western blotting was used to detect the change in protein expression of the MAPK signaling pathway, including the extracellular regulated kinase (ERK1/2), c-Jun N-terminal kinase (JNK), and p38 kinases as well as their phosphorylated forms. Although p-JNK and p-p38 had no difference between cisplatin and the combined treatment in SKOV3 and SKOV3/DDP cells (Figure 5A), p-ERK was obviously induced by cisplatin, while it was inhibited in the combined treatment in both SKOV3 and SKOV3/DDP cells (Figure 5A). As a kinase, ERK is known to phosphorylate a series of downstream target proteins to mediate cell survival (Salaroglio et al., 2019). To verify the role of ERK signaling in the combined treatment, LM22B-10, an ERK agonist, was used (Yang et al., 2019; Huang et al., 2021). First, we determined the optimal concentration and action time of LM22B-10. SKOV3 and SKOV3/DDP were incubated with different concentrations of LM22B-10 for 6 h, and the cell viability was determined after 24 h by the CCK-8 assay. The results showed that concentrations of no more than 20 μM of LM22B-10 are non-toxic for SKOV3 and 50 μM for SKOV3/DDP at 24 h (Supplementary Figure S5A). Then, the non-toxic concentrations of LM22B-10 were chosen and further tested. The result showed that pretreating 20 μM LM22B-10 for 6 h effectively activated ERK signaling both in SKOV3 and SKOV3/DDP treated with GAD + cisplatin (Supplementary Figure S5B). Next, we performed CCK-8 and Annexin V/PI staining to test cell viability and death of the combined treatment with or without LM22B-10. The results showed that pretreating SKOV3 and SKOV3/DDP cells with 20 μM LM22B-10 for 6 h could partially increase cell viability (Figure 5B). Likewise, LM22B-10 pretreatment also reduced the apoptosis rate in SKOV3 and the necrosis rate in SKOV3/DDP cells (Figures 5C–E). Such an observation thus indicates that the combined effects of GAD and cisplatin in SKOV3 and SKOV3/DDP are mediated by ERK signaling inhibition. Furthermore, to identify the relationship between enhanced ROS production and impaired ERK activation, we measured the p-ERK in the combined treatment complementing with NAC and found that NAC significantly enhanced the p-ERK in both SKOV3 and SKOV3/DDP cells with the combined treatment of GAD and cisplatin (Figure 5F). Such results thus support our notion that ERK inhibition in the combined treatment is mediated by oxidative stress. Finally, to explore whether SBSGL promoted cisplatin sensitization of ovarian tumors through the ROS-ERK pathway *in vivo*, DHE staining and Western blotting were used to test ROS and p-ERK levels in tumor tissues, respectively. The results of DHE staining showed that the combined treatment group had higher fluorescence intensity than cisplatin treatment, implying a higher level of ROS in the combination treatment group (Figure 5G). In addition, p-ERK/ERK in the tumor tissue lysate was significantly increased by cisplatin treatment, while it was reduced by the combined treatment (Figure 5H). The above results thus indicate that combining SBSGL or GAD with cisplatin could induce oxidative stress, leading to the inhibition of p-ERK signaling, and eventually suppression of cell proliferation and promotion of cell death in ovarian tumors (Figure 6).

## Discussion

Ovarian tumor is one of the most fatal gynecological malignancies around the world. Although some novel treatment strategies are emerging in preclinical studies, platinum-based chemotherapy is still the standard first-line treatment for advanced ovarian tumors (Lheureux et al., 2019). Cisplatin is one of the most important platinum drugs. However, drug resistance and severe adverse effects limit its clinical application (Ushijima, 2010). Therefore, enhancing cisplatin sensitivity and alleviating its side effects are important to help delay tumor progression and improve prognosis in ovarian cancer patients.

SBSGL is processed from the traditional medicinal mushroom *Ganoderma lucidum*. During the past decades, the antitumor effects of SBSGL have been tested in different cancer models (Wang et al., 2015; Sang et al., 2021; Shi et al., 2021). For instance, Zhao et al. showed that SBSGL could inhibit ovarian cancer cell growth by regulating cell cycle and apoptosis (Zhao et al., 2011). However, the synergistic effect of SBSGL and cisplatin on ovarian tumors and their combined toxicity still needs to be evaluated *in vivo*. Therefore, we constructed the ovarian tumor xenograft nude mouse model to examine the combined effects of SBSGL and cisplatin. In our study, tumors treated with SBSGL and cisplatin exhibited lower expression of Ki-67 and a higher apoptosis rate relative to the cisplatin treatment, thus demonstrating the sensitization effect of SBSGL on cisplatin in ovarian cancer.

The common adverse effects of cisplatin include nephrotoxicity, gastrointestinal toxicity, and myelosuppression (Ghosh, 2019). In the present study, we found that SBSGL neither aggravated nor protected against cisplatin-induced nephrotoxicity. Importantly, SBSGL could obviously reduce cisplatin-induced intestine damage, suggesting that SBSGL has a protective effect against cisplatin-induced intestinal toxicity. This result was consistent with an earlier report that polysaccharide extracted from spores of *Ganoderma lucidum* could ameliorate paclitaxel-induced intestinal injury (Li et al., 2020). For bone marrow suppression, cisplatin-induced myelosuppression often presents as anemia, leukopenia, and thrombocytopenia in clinics (Oun et al., 2018). In our nude mouse models, cisplatin treatment mainly induced megakaryocyte dysfunction along with a reduced number of platelets, without any obvious cytotoxic effect on the granulocytes and erythrocytes. Species differences might contribute to this different response of marrow cells to cisplatin. Meanwhile, we also found a larger proportion of megakaryocytes in the bone marrow of the control group than in a normal healthy person. This result was partially consistent with Sonali Sinha’s finding that megakaryocytes were the most affected blood cells by the cisplatin treatment (Sinha et al., 2015). To conclude, our results proved that combining SBSGL with cisplatin could reverse cisplatin-induced intestinal injury, megakaryocyte suppression, and loss of platelets, without having a protective effect on nephrotoxicity.

With the development of phytochemical techniques, more and more monomer components of SBSGL are being extracted, and their therapeutic value is waiting to be further tested. Among them, GAD, one of the main components of triterpenoids in SBSGL, has been reported to possess antitumor activity (Liu et al., 2018; Shao et al., 2020). However, the effect of GAD on ovarian tumors is still unknown. Herein, our data showed that GAD alone (up to 200 μM) had no obvious cytotoxicity to SKOV3 and SKOV3/DDP cells. Similar results were reported in colon cancer (Liu et al., 2018). In addition, we also found that the combined treatment of GAD and cisplatin induced apoptosis in SKOV3 cells, while apoptosis and necrosis were induced SKOV3/DDP cells. The different types of death might attribute to the different characters of cisplatin-sensitive and cisplatin-resistant ovarian cells. Cisplatin-resistant tumor is characterized by apoptosis resistance and high expression of various anti-apoptotic/pro-survival proteins such as Bcl-2 and survivin (Galluzzi et al., 2012; Wang et al., 2020). As a result, the cisplatin-resistant SKOV3/DDP cells were more inclined to necrosis than apoptosis.

ROS is an important mediator in signaling cascades, regulating various cellular events, including proliferation, differentiation, and apoptosis (Moloney and Cotter, 2018). Previous studies proved that cisplatin’s cytotoxicity largely depended on the accumulation of ROS and antioxidants could reverse cisplatin-induced apoptosis (Ma et al., 2014). Therefore, we explored whether ROS participated in the synergistic effect of GAD on cisplatin. We found that the combination of GAD and cisplatin obviously increased intracellular ROS in both SKOV3 and SKOV3/DDP cells. More importantly, the addition of an antioxidant like NAC could significantly reverse the effects of the combined treatment on cell proliferation and death. These results support the notion that the sensitization effect of GAD on cisplatin is most likely mediated *via* elevated intracellular ROS levels. Furthermore, we also found that although GAD increased the level of ROS only in SKOV3/DDP cells, it did not affect the cell viability and death of SKOV3/DDP. This might be attributed to several reasons that cisplatin-resistant ovarian tumors have high expression of antioxidant proteins like H-Ferritin (Salatino et al., 2019) and signaling such as the Nrf2 pathway (Xia et al., 2014) to ameliorate oxidative stress. In addition, as mentioned above, cisplatin-resistant ovarian tumors are more apoptosis resistant and have higher expression of pro-survival proteins.

Consisting of ERK1/2, JNK, and p38 kinases, the MAPK pathways regulate various cellular events, including cell survival, apoptosis, and stress response (Achkar et al., 2018). These phosphorylation cascades could work either upstream or downstream of ROS (Jalmi and Sinha, 2015). It has been well established that activation of ERK is a critical mediator in cisplatin resistance in ovarian tumors. For instance, cisplatin could induce the activation of ERK1/2 in SKOV3 and inhibiting ERK1/2 activity with PD98059 could enhance cisplatin cytotoxicity (Cui et al., 2000). In addition, p-ERK promoted cisplatin resistance in ovarian tumors through stabilizing HIF-1α by phosphorylating PHD2, an enzyme hydroxylating HIF-1α and inducing its degradation by a ubiquitin-mediated pathway (Li et al., 2019). In the current study, an ERK agonist, LM22B-10, was able to reactivate p-ERK and subsequently reverse the sensitization effect of GAD on cisplatin in the ovarian tumor. Therefore, we conclude that ERK signaling inhibition mediated the sensitization effects of GAD on cisplatin in the ovarian tumor. Moreover, we further clarified that ERK phosphorylation inhibition in the combined treatment was regulated by increasing the ROS level since reduced p-ERK level in the combined treatment group could be restored by ROS scavenger, NAC. To conclude, our research demonstrated that the combined treatment of GAD and cisplatin increases the intracellular level of ROS, inhibits ERK phosphorylation activation, and subsequently suppresses cell proliferation and promotes cell death in the ovarian tumor. However, the potential mechanism of ROS-mediated regulation of ERK phosphorylation in ovarian tumors needs to be explored in future studies. Meanwhile, the therapeutic value of GAD also needs to be further verified *in vivo*.

## Conclusion

In summary, our present study proved that SBSGL could enhance the antitumor effect of cisplatin on ovarian tumors and attenuate cisplatin-induced intestinal damage and myelosuppression *in vivo*. Furthermore, we clarified that GAD, one of the main components of SBSGL, contributed to the cisplatin sensitization in the ovarian tumor. The mechanism was related to the increased ROS followed by the inhibition of the ERK signaling pathway. Conclusively, our study suggests that SBSGL is beneficial to ovarian cancer patients under cisplatin chemotherapy and that GAD is a promising component that deserves further development and clinical verification.

## Data Availability

The raw data supporting the conclusion of this article will be made available by the authors, without undue reservation.
