# Supplementary material for: Sporoderm-Broken Spores of Ganoderma lucidum Sensitizes Ovarian Cancer to Cisplatin by ROS/ERK Signaling and Attenuates Chemotherapy-Related Toxicity
Source: Front Pharmacol. 2022 Feb 21;13:826716. doi: 10.3389/fphar.2022.826716 (PMC8900012; doi:10.3389/fphar.2022.826716)
Supplement: Supplementary file 1 [file DataSheet1.docx]

Supplementary Material

**
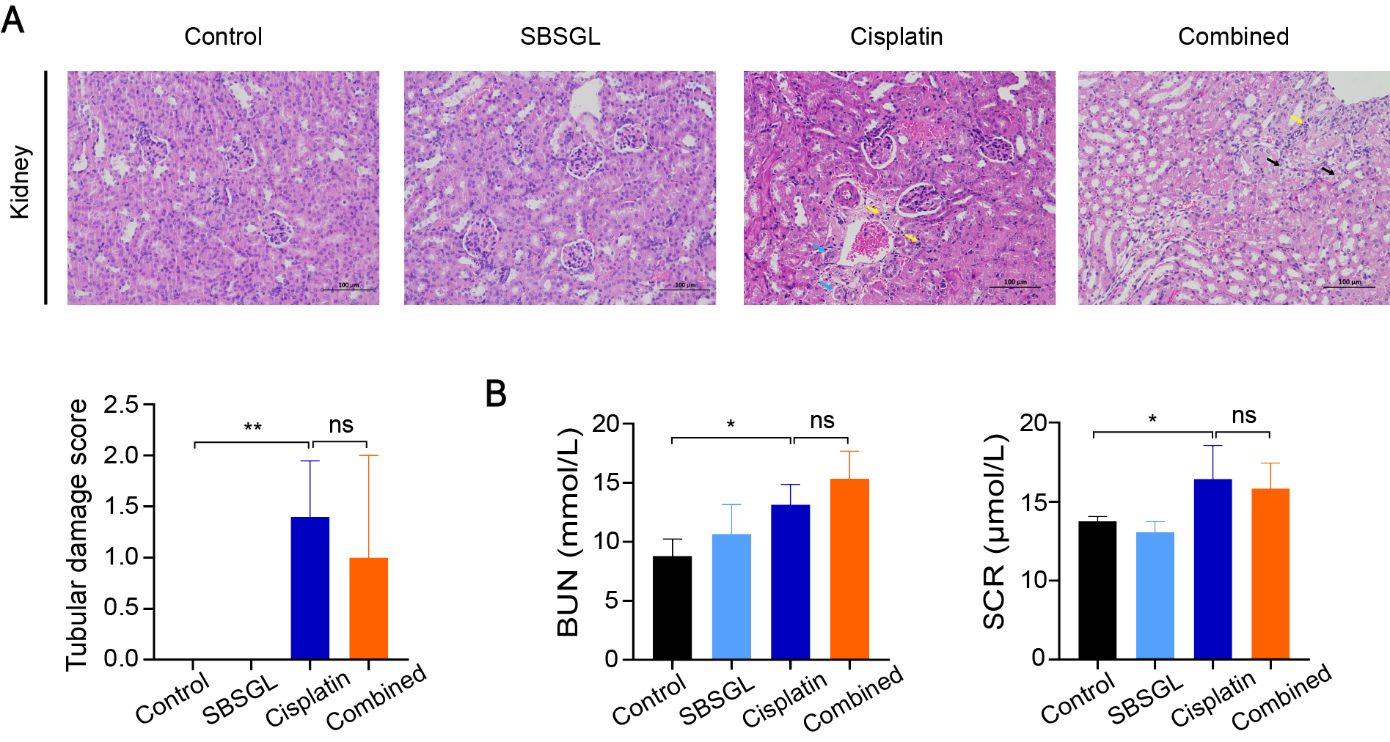
**

**Supplementary Figure 1.** Sporoderm-broken spores of Ganoderma lucidum (SBSGL) had no obvious protective effect on cisplatin-induced nephrotoxicity. **(A)** The histological features of kidneys and tubular damage score was quantified. Arrows indicate infiltration of inflammatory cells (yellow), tubular atrophy (blue) and edema (black) respectively. **(B)** The level of BUN and SCR in serum of nude mice. BUN: blood urea nitrogen; SCR: serum creatinine. Data are presented as the mean ± SD (n=5). ^**^p< 0.01, ^*^p< 0.05, ns, no statistical significance.


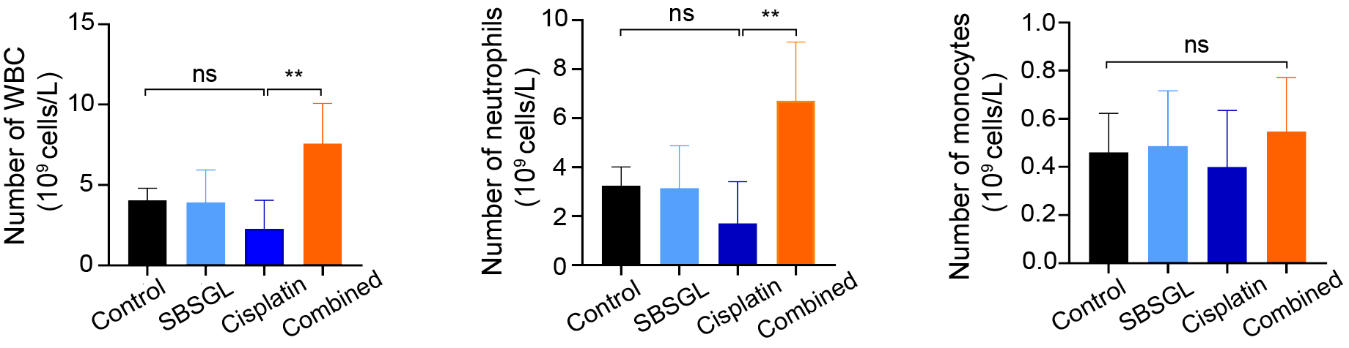


**Supplementary Figure 2.** Cisplatin and SBSGL alone and their combination didn’t affect the number of WBC, neutrophils and monocytes in the blood of nude mice. WBC: white blood cells. Data are presented as the mean ± SD (n=5). ^**^p< 0.01, ns, no statistical significance.


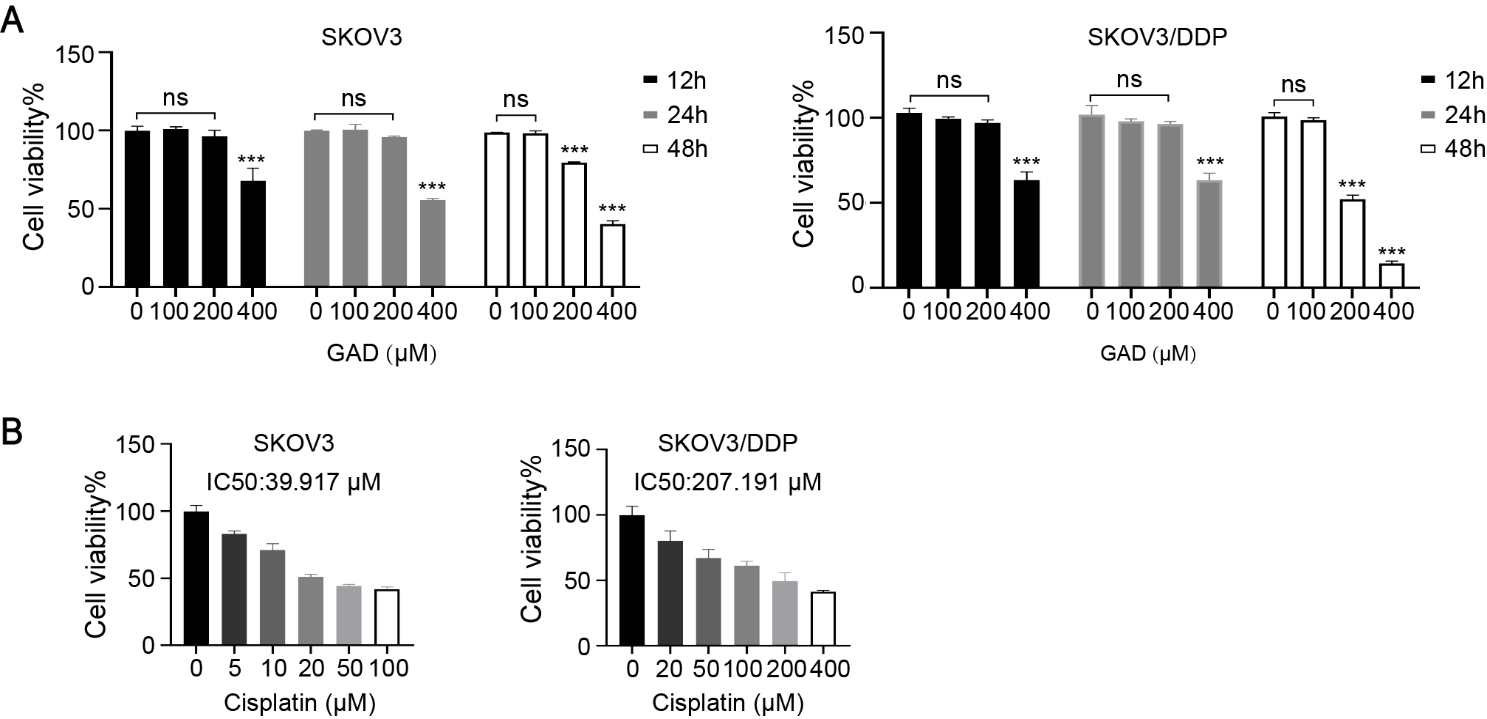


**Supplementary Figure 3.** The cell viability of SKOV3 and SKOV3/DDP treated with ganoderic acid D (GAD) and cisplatin alone. **(A)** The cell viability of SKOV3 and SKOV3/DDP incubated with indicated GAD (100, 200, 400μM) for 12, 24 or 48h. **(B)** SKOV3 and SKOV3/DDP were treated with indicated cisplatin respectively for 24h followed by cell viability detection. The IC50 values of cisplatin were calculated by SPSS 25.0. GAD: ganoderic acid D. Data are presented as the mean ± SD (n=3). ^***^p< 0.001, ns, no statistical significance.


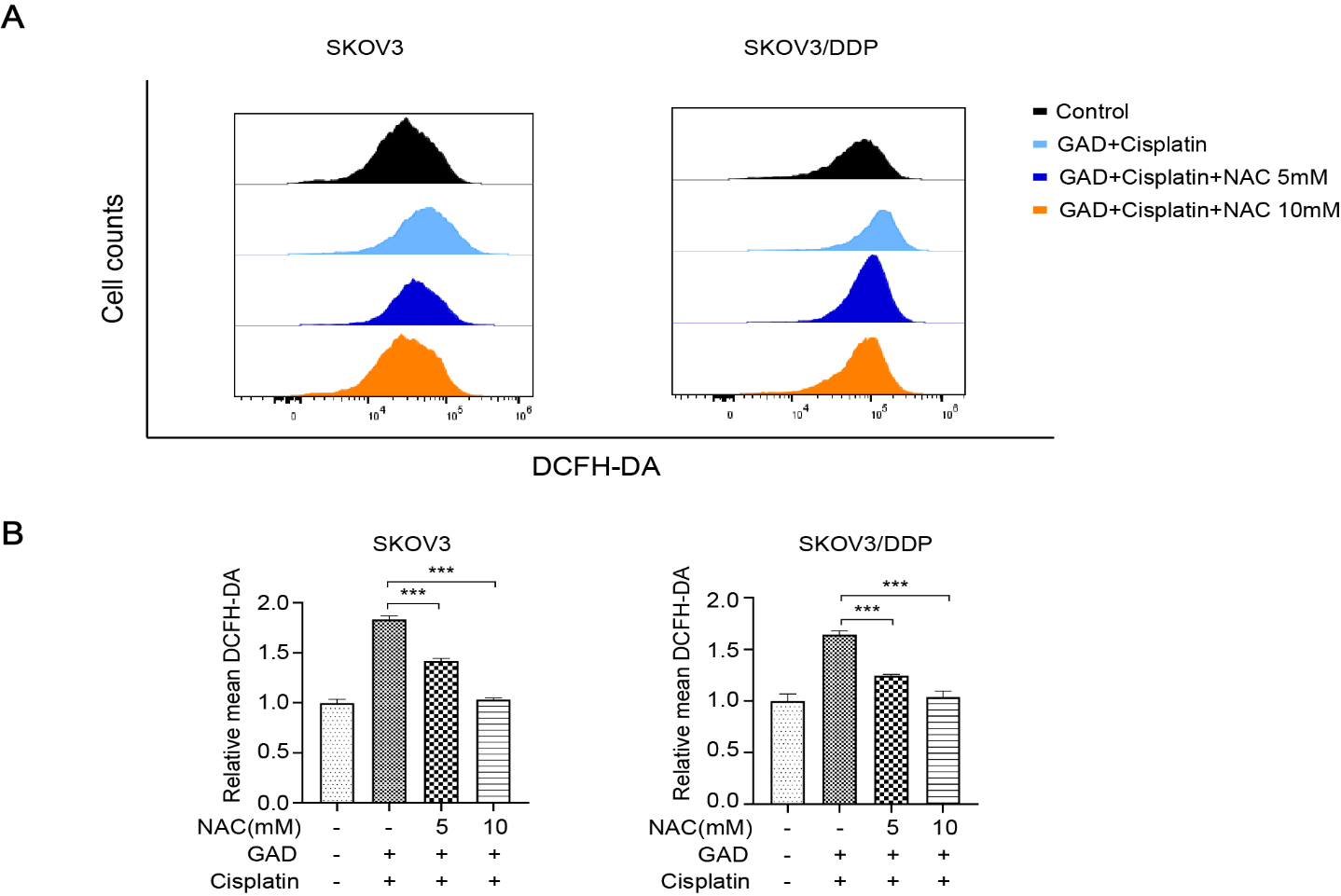


**Supplementary Figure 4.** The intracellular ROS level of SKOV3 and SKOV3/DDP. **(A)** SKOV3 and SKOV3/DDP were incubated with the combination of GAD and cisplatin with or without different concentrations of NAC (5mM and 10 mM) for 24h followed by ROS detection using DCFH-DA probe. **(B)** The bar plots of the mean fluorescence intensity of DCDH-DA in SKOV3 and SKOV3/DDP. Data are presented as the mean ± SD (n=3). ^***^p< 0.001.


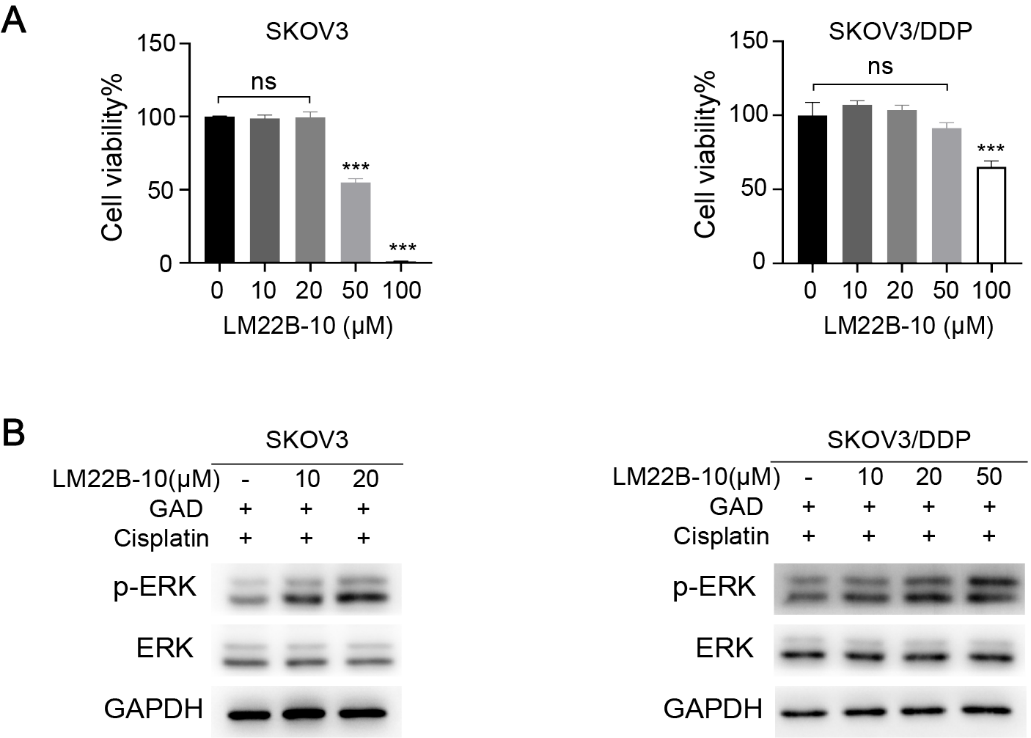


**Supplementary Figure 5.** The optimal concentration and action time of LM22B-10. **(A)** SKOV3 and SKOV3/DDP were incubated with different concentrations of LM22B-10 (10,20,50,100 μM) for 6h followed by cell viability detection after 24h. **(B)** The protein expression of p-ERK in SKOV3 and SKOV3/DDP. Data are presented as the mean ± SD (n=3). ^***^p< 0.001, ns, no statistical significance.
